# Internet of Things Based Contact Tracing Systems

**DOI:** 10.3390/s21217124

**Published:** 2021-10-27

**Authors:** Peng Hu, Philippe Lamontagne

**Affiliations:** Digital Technologies Research Center, National Research Council of Canada, Ottawa, ON K1A 0R6, Canada; philippe.lamontagne2@nrc-cnrc.gc.ca

**Keywords:** contact tracing, Internet of Things, privacy, proximity sensing, environment sensing, SARS-CoV-2, COVID-19, peer to peer, object to peer, transmission methods

## Abstract

The COVID-19 pandemic has significantly threatened the health and well-being of humanity. Contact tracing (CT) as an important non-pharmaceutical intervention is essential to containing the spread of such an infectious disease. However, current CT solutions are fragmented with limited use of sensing and computing technologies in a scalable framework. These issues can be well addressed with the use of the Internet of Things (IoT) technologies. Therefore, we need to overview the principle, motivation, and architecture for a generic IoT-based CT system (IoT-CTS). A novel architecture for IoT-CTS solutions is proposed with the consideration of peer-to-peer and object-to-peer contact events, as well as the discussion on key topics, such as an overview of applicable sensors for CT needs arising from the COVID-19 transmission methods. The proposed IoT-CTS architecture aims to holistically utilize essential sensing mechanisms with the analysis of widely adopted privacy-preserving techniques. With the use of generic peer-to-peer and object-to-peer sensors based on proximity and environment sensing mechanisms, the infectious cases with self-directed strategies can be effectively reduced. Some open research directions are presented in the end.

## 1. Introduction

The recent COVID-19 pandemic has caused extensive interruptions to the world and threatened human health and well-being. In search of ways to combat it and its new variants, with the absence of effective vaccination and treatments, non-pharmaceutical interventions (NPIs) [1] aiming to reduce contact rates and tracking suspected exposure to an infectious disease are essential to the containment of emerging epidemics [2]. Contact tracing (CT) and Internet-based systems have provided important information for the previous influenza pandemics [3] and for the current COVID-19 pandemic. Internet of Things (IoT) technologies have provided great promises for enabling advanced sensing, communication, and computation for CT solutions, but there are still many challenges to overcome.

Prior to COVID-19 pandemic, CT solutions have been centered on influenza-like diseases, where peer-to-peer (P2P) event data collected from sensors and Internet-based social networking tools on mobile devices [4,5] are employed to study the outbreaks of infectious diseases based on physical interactions between individuals. Many current smartphone-based CT apps for COVID-19 using one or more built-in sensing capabilities, such as GPS, cellular, and Bluetooth, have been available from public health agencies [6] and 3rd-party providers [7,8]. Statistical evaluation using a smartphone for CT has shown its effectiveness in [9], where a stochastic model was developed considering the CT options on a smartphones with the analysis of quarantine measures. Extending the sensing options to the IoT context following an IoT-CTS architecture [10] provides a more generic scope of CT applications. The state-of-the-art in the literature has validated the effectiveness and modeling of an CT application, there are still challenges to be resolved. However, the current CT solutions primarily focus on the proximity and positioning sensing for P2P contact events between individuals, where these sensing technologies are assumed to be used by individuals. A wide scope of sensing technologies that can not only detect the P2P contact events but also detect contact events between objects and peers, i.e., object-to-peer (O2P), have hardly been addressed. Furthermore, the use range of sensing technologies and their uses for CT, heterogeneity, and system dynamics bring many challenges to design, analysis, and evaluation for an IoT-CTS solution.

This paper aims to answer the fundamental question on “what and how IoT sensors can be used in an IoT-CTS?” based on the IoT-CTS solutions. We envision a generic architecture for IoT-CTS design, evaluation, and deployment. This paper also investigates the important aspects of privacy preservation and P2P/O2P sensing within the IoT-CTS architecture, where the real-world factors that are related to the actual performance of an CT solution but difficult to analyze in a theoretical model. In summary, the main contributions of this paper are as follows:

- A detailed design of an IoT-CTS system and the operational models for supporting privacy preservation is discussed;
- The applicable sensors based on proximity and environment sensing are discussed in our agent-based simulations;
- Based on our case study, the measure options using an IoT-CTS for disease containment are suggested.

The remainder of the article is structured as follows. In Section 2, we examine the principles and enabling technologies behind the IoT-CTS and address challenges. By overviewing the key topics, such as sensing technologies, data, architecture, protocol stack, and privacy, an IoT-CTS architecture is proposed in Section 3. Section 4 discusses the privacy considerations for the proposed IoT-CTS architecture. Section 5 represents the case study supported with agent-based simulations. Finally, in Section 6 we pinpoint the open research directions regarding an IoT-CTS.

## 2. Related Work

### 2.1. Theoretical Grounds in Epidemiology

The classical SIR model provides a general explanation of why an IoT-CTS can work. The typical reproduction number derived from the SIR model is proportional to the effective contact rate while the recovery rate is kept the same. An IoT-CTS can intervene in the transmissions of an infectious disease based on at least two factors: (a) the time required to transition from the susceptible state (S) to the infectious state (I) is shortened, as the individual risk of exposure to infection through the effective CT is promptly handled by IoT-CTS; (b) responsive self-directed strategies can be taken as soon as the exposure risks to the potential infections are available. The strategies can result in actions, such as reduced interactions between individuals.

### 2.2. Interacting Networks and CT Modeling

Some recent related work in epidemiological modeling has been focused on interacting networks. Farrahi et al. [11] studied an approach to reconstructing physical interactions from text messages, Bluetooth module activities, and communication logs on a mobile phone. In [11], the communication networks and physical interaction networks are modeled in a dual network setting, where the communication networks are viewed as proxies of the physical interaction networks. Zuzek et al. [12] discussed the isolation effect based on the SIR model following a two-layer network, where one layer is in the work environment and the other layer is in the social environment. Salathé et al. [3] suggested employing Internet-based systems for surveillance can provide “important early epidemic intelligence” for past pandemics, such as the 2003 SARS and 2009 H1N1. Using non-public health data for epidemiological research has emerged under the umbrella of digital epidemiology. The theoretical modeling for agent-based CT based on the susceptible, exposed, infected, and recovered (SEIR) epidemiological model was recently proposed in [13], where the trajectories are modeled as a stochastic process. However, such a model only considers a few parameters in addition to the SEIR model, such as the CT rate, success rate of CT, and average daily contacts. The susceptible–exposed–infectious–confirmed–recovered (SEICR) model is adopted in [14] to the agent-based modeling (ABM) as an alternative to the theoretical modeling to deal with the complexity of the related factors. However, the agent-based modeling or simulation for applicable sensing mechanisms arising from COVID-19 with an IoT architecture has not been studied in the literature.

### 2.3. Existing CT Applications with Partial Sensing Capability

CT applications have been used in the COVID-19 and past pandemics, where sensing mechanisms play a key role in generating proximity data required on user devices. QR code scanning is assisted with a manual process, so its speed is considered slow. Detecting a touch event for O2P can be through touch or proximity sensing, based on radio-signal strength (RSS), inductive, capacitive, ultrasonic, or photoelectric mechanisms. Proximity sensing can be derived from RSS values with the use of existing wireless networking technologies, such as radio-frequency identification (RFID), including near-field communication (NFC), and a wireless personal-area networking (WPAN) technology, including Bluetooth/BLE and IEEE 802.15.4 [5,11]. Bluetooth/BLE proximity sensing has been adopted by the COVID-19 CT apps in most countries [15]. RFID sensors can detect touch and proximity events in a fast and high-precision manner. The biosensor [16] based on plasmonic sensing represents a range of biosensors that can detect the novel coronavirus (SARS-CoV-2) in an ambient environment. Such a biosensor has a higher speed than the clinical tests but usually slower than non-biosensors. In general, a sensor may need extra modules for broadcasting or exchanging identifiers or messages with IoT-CTS entities.

Some sensing-based CTS have emerged in the recent decade. The spatial-proximity interaction data have been studied in determining the spread of infectious diseases. Génois et al. [17] used different proximity data of individuals, such as face-to-face proximity and workplace interaction data with high-resolution time-resolved datasets. Jeong et al. [4] suggested a solution using magnetometers on smartphones based on magnetic induction, where magnetometer readings on smartphones are used to determine proximity between the users. A P2P CT app called “TrackCOVID” has been developed [7] with privacy preservation. The spatial-proximity information can infer the influenza infection pattern based on sumptuous data with the Bluetooth scanning and Wi-Fi in the PocketCare mobile app [5]. A new Bluetooth low-power (BLE) specification for contact detection services enabled by Apple and Google has become available in mobile devices with Bluetooth modules during the COVID-19 pandemic. The PrivateKit mobile app developed by MIT uses Bluetooth and GPS trails for CT with some privacy considerations. These CT apps are implemented without following a management architecture and sufficient modeling for evaluating the performance. However, due to the prevalent uses of the BLE-based COVID CT apps in most countries [15], we will adopt the proximity sensing mechanism as a benchmark scenario in our evaluation.

## 3. Proposed Solutions to IoT-CTS

**Figure 1:**
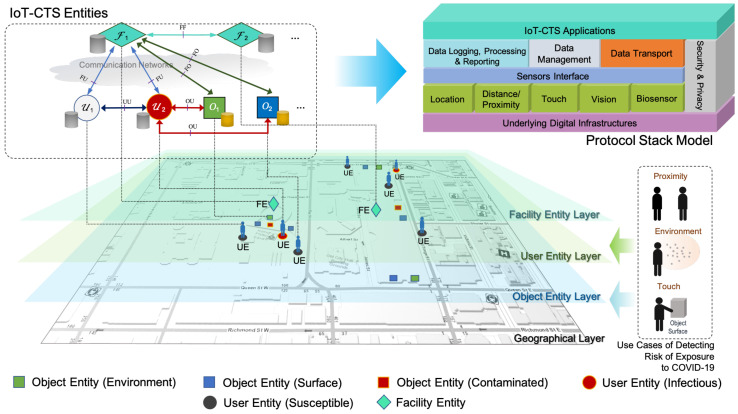
An IoT-CTS architecture.

In this section, we will propose the IoT-CTS architecture as shown in Figure 1. We will analyze the categories of applicable sensing technologies, followed by their integration into the architectural elements into the proposed protocol stack. The conceptual layers where different elements function, as well as the interaction models, will be discussed.

### 3.1. Sensing Technologies for IoT-CTS

Effective use of sensing technologies in an IoT-CTS requires us to understand the applicable sensors and essential features for detecting contact-related events. For COVID-19, it is currently known [18] that close contacts between individuals (collectively referred to as P2P) through respiratory droplets and contacts events between individuals and contaminated objects or surfaces (collectively referred to as O2P) are the main transmission methods. The object here is defined to include an object, an object surface, or a surrounding environment with coronavirus aerosols or particles in the air. The sensing technologies for IoT-CTS are therefore required to detect contact events from P2P/O2P interactions.

A list of sensors categorized in various types is shown in Table 1, where their basic characteristics are presented, including their detection capability for P2P/O2P events and other factors, where “Automatic” indicates whether a sensor can be operated automatically or manually; “Speed” means the time it takes for a sensor to obtain the required data for CT; “Precision” means the precision quality for detecting an event. Location-based sensors can provide proximity data between individuals. The distance sensors can provide high-precision ranging capability between individuals or objects. Computer-vision sensors, such as cameras can infer proximity, gesture, or interactions between individuals or objects, together with additional information, such as body temperatures. At the same time, they normally take more compute power and time than other sensors.

A sensing system for IoT-CTS may use multiple types of sensors. For example, a sensing system deployed on an entrance may include multiple sensors, such as a biosensor for monitoring coronavirus in the ambient environment, a capacitive sensor for monitoring touch events and triggering the sanitization procedures, and a BLE device for proximity sensing and networking.

The sensing technologies discussed here are mainly based on the transmission methods of the infectious disease arising from the COVID-19 pandemic and similar influenza pandemics, such as the H1N1 pandemic in the past and its possible variants in the future. Furthermore, as sensing technologies are expected to keep evolving in terms of sensing accuracy and capability to detect additional viruses, we need a stable infrastructure for operating and managing all applicable sensors for such pandemics. One way of resolving this challenge is to design an IoT-CTS architecture that can provide a holistic architecture for existing and future sensors operations. The design of such an IoT-CTS is discussed in the subsequent section.

### 3.2. Sensing Data

There are various kinds of data that can be obtained from various sources, such as sensors, IoT devices, and social media. The generation and use of these data is mainly dependent on the sensing technologies employed. The essential features of the sensing data, such as identifier, location, and timestamp, are important to IoT-CTS solutions. The identifier feature is the unique index of a user/object, which should be anonymized. The location feature represents coordinates of a user/object in a geographic coordinate system. Additional features can be used, such as an S/I/R state of a user/object, indicating the basic attributes from the SIR model.

The availability of spatial-temporal sensing data collected for each entity allows us to log the exposure risks, study the transmission patterns over time, and take actions on the contaminated environments or objects. Furthermore, the granularity of sensing data is determined by the application requirements. For example, if we deploy an IoT-CTS in a public facility (as illustrated in Figure 1), where contact events can be tracked in an area. Once the risk of exposure to the coronavirus is detected, users can be notified quickly.

### 3.3. An IoT-CTS Architecture

An IoT-CTS architecture [10] can deal with heterogeneity and complexity, and well integrate sensing components, sensing data, connectivity options, and computation methods. This is one distinctive characteristic compared to the existing CTS solutions.

A layered view is shown in Figure 1, where we can see the architecture contains the geographical layer, object endpoint (OE) layer, user endpoint (OE) layer, facility endpoint (FE) layer, and IoT-CTS application layer. With the layered view, use cases of detecting risks of exposure to COVID-19 based on different sensing mechanisms for P2P/O2P contact events can be handled. A geographical layer refers to a geographical area where an IoT-CTS is deployed for CT tasks. The proposed architecture has three basic entities: UE, OE, and FE. An OE is an IoT-CTS endpoint on for monitoring an object or in an environment, and OE is able to connect to various sensors through sensors interfaces, as shown in the *protocol stack model* of Figure 1. The object refers to an object or an object surface being monitored, while the environment refers to a space being monitoring. A UE is the IoT-CTS endpoint on a user device interfacing with applicable sensing devices, as shown in Table 1. An FE is an endpoint FE is located at a local or remote facility that can interact with UEs or OEs.

The block diagram of a generic protocol stack model for an IoT-CTS is depicted in Figure 1 which includes several key modules. To make the IoT-CTS application endpoints be able to interface with various sensor hardware components, a *sensors interface* is needed. The *data logging, processing and reporting* module handles the monitoring, processing, and reporting of sensing data, while the *data management* module tackles data queries, administration, and storage, in compliance with the settings in the *security and privacy* module. The *data transport* module deals with data transfers and interactions with an external endpoint securely and effectively. The underlying digital infrastructure can provide security services, transmission services, and computing services, provided by edge computing nodes, local networks, etc.

An IoT-CTS user needs to run UE on the user device, which can interact with UE or FE, depending on an interaction model. All entities are connected to a data storage for the key functional blocks shown in the *protocol stack model*. An FE can be deployed in a building or facility through communication networks managed by a data center or public health authority to handle the data compliance with security and privacy considerations. In addition, FEs can be implemented in a centralized or distributed manner. In a centralized implementation, an FE can be a public health agency that usually manages reported cases and CT data. In a distributed implementation, a local FE entity deployed at a public facility, such as a plaza, museum, or library, can be implemented, where local computation, data transfer and storage, and decisions can be made. All distributed FEs can be hierarchically federated into a centralized entity, and parent FE entity can be public health agency managing the data from distributed FEs.

The proposed architecture can operating at the application layer, independent of the existing transport protocols, such as cellular, Wi-Fi, and low-power wide-area (LPWA) networks. This ensures minimum changes to the underlying digital infrastructure and maximum compatibility and scalability.

### 3.4. Interactions between UEs, OEs, and FEs

The communication interactions between architectural entities, FE, UE, and OE are depicted in Figure 1, where the interfaces named “OU”, “UU”, “FU”, and “FO” refer to the interactions of OE-UE, UE-UE, FE-UE, and FE-OE, respectively. Each interface can be operated in a uni- or bi-directional manner. It is worth noting that these interfaces can be optionally used and how much they are used is dependent on the application requirements. The interfaces may be used for device or data management purposes.

Let us discuss generic interaction models following the P2P and O2P CT tasks. Interactions between UEs and FEs can be performed in three basic models: centralized, user-centered, and distributed. In a centralized model, two UEs can detect the proximity events between each other and report the event data to an FE over a communication network. In this model, an FE has the full information and it is responsible for handling data access requests from UEs, and enable them to retrieve data that are authorized for the user to view through an authentication process. Example data access requests authorized for a user include retrieval of exposure risk of infection at a certain time period and area, management of infection self-reports, individualized public health guidelines, etc. In a user-centered model, a UE can detect and log contact events with other UEs, and to retrieve information from an FE. UEs can exchange data in a distributed model and assess user’s infection risks locally, while a UE may transmit data to the FE and retrieve data from it. The data storage used on the entities can be any standard relational database system supporting data encryption.

Due to the frequent interactions between a UE and an OE, it is necessary to further analyze the interaction models between them. Interactions between a UE and an OE can be performed in two models: direct and indirect. The direct model means the contact event data are stored in both UE and OE. To save computing resources, the OE can choose to transmit these data to an FE. The indirect model means the contact event data between a user and an object are stored via a UE, which can opt to transfer data to the FE. The interaction models can lead to variants of IoT-CTS setups. For example, in a lightweight IoT-CTS application, a UE may run on a mobile device equipped with sensing devices, and an OE involves no processing and storage of data and only transfers data to an FE or a UE.

### 3.5. Design Decisions

The proposed IoT-CTS architecture can be used to design an IoT-CTS application, including the application endpoints of UE, OE, and FE. The scalability is one of the major design considerations, where the IoT-CTS application can support different scenarios at scale. In a small-scale CT scenario, the IoT-CTS application can be deployed to monitor a local area, and the FE is running at an on-site central server to manage data transfer, storage, processing, logging, and reporting from UE and OE endpoints. The transport of the underlying digital infrastructure in this scenario is likely to be a local-area network where all architectural entities are connected. In a large-scale CT scenario, the IoT-CTS application is deployed to monitor multiple geographical areas. Each area can follow the aforementioned small-scale setup with one local FE. Multiple local FEs will be federated into a central FE managed by a public health authority, where the FF interface is used to exchange messages between local and central FEs. The transport networks as underlying infrastructures can vary as long as they support the Internet protocol suite.

The IoT-CTS entities can operate with fault-tolerant consideration where service downtown should be minimized. First, all endpoints need to back up the latest state of the operation, and the frequency of such a backup operation is up to application configuration. The data on the device need to be stored in a secure environment with encryption which is managed through the *security and privacy* module. When application errors happen, all endpoints can load the saved operation state and resume operation. Additional generic fail-safe deployment strategies using redundant sensors and applicant instances can be considered on FE and OE to ensure continued operations. When using computing resources for IoT-CTS application development, fault-tolerant capabilities can be achieved at the platform level, where instance replacement, load balancing, and high availability strategy can be utilized.

Although the IoT-CTS architecture can enable sensing, data management, connectivity, and computation for various CT tasks, there are various factors related to a solution design. As privacy is an important consideration for CTS solutions, we will analyze this topic following the proposed architecture-centered interaction models in the next section. In addition, configuration parameters that may affect the performance will be discussed in the case study.

## 4. Privacy Considerations for IoT-CTS

CT and privacy may have different goals, nevertheless many systems have been proposed to facilitate digital CT, while ensuring that the users’ privacy is preserved as much as possible. In this section, we present the main privacy goals for CT systems, how the most widely adopted proposals for protecting privacy in CT systems operate, and how they can fit in an IoT setting.

The privacy goals of CTS are:

- **Location Privacy.** The CTS should not leak information on the location history of its users;
- **Social Privacy.** The CTS should not leak information on the social graph of its users;
- **Anonymity/Pseudonymity.** It should not be possible to determine if a user was diagnosed positively.

Most of the widely discussed CT solutions employ a similar concept. For example, the DP-3T, ROBERT, PEPP-PT and NTK CT frameworks, and the joint Apple/Google CT technology all follow this approach. Each device broadcasts an *ephemeral identifier* that it updates at specific time intervals (usually 15 min). Each device also listens to other devices’ identifiers and stores them under some conditions (e.g., minimal duration and strength of signal). Each device thus maintains two lists of identifiers: its own broadcast identifiers and its received identifiers (i.e., registered contacts). Old identifiers are routinely removed from both lists. Once a user is diagnosed positive, it is granted the permission to upload information to a server and any contacts are alerted of the potential risk.

- In *centralized* CT, the server provides the identifiers for each user. When a user is diagnosed, it uploads its list of registered contacts to the server. Transmission risk is computed by the server: it alerts each user that has a broadcast identifier that was captured by the diagnosed user;
- In *decentralized* CT, the diagnosed user uploads the list of its own broadcast identifiers to the server. Transmission risk is computed by the users: they download the list from the server and compare it against their own list of received identifiers.

For a comprehensive overview of centralized versus decentralized CTS, we refer the reader to [19].

**Figure 2:**
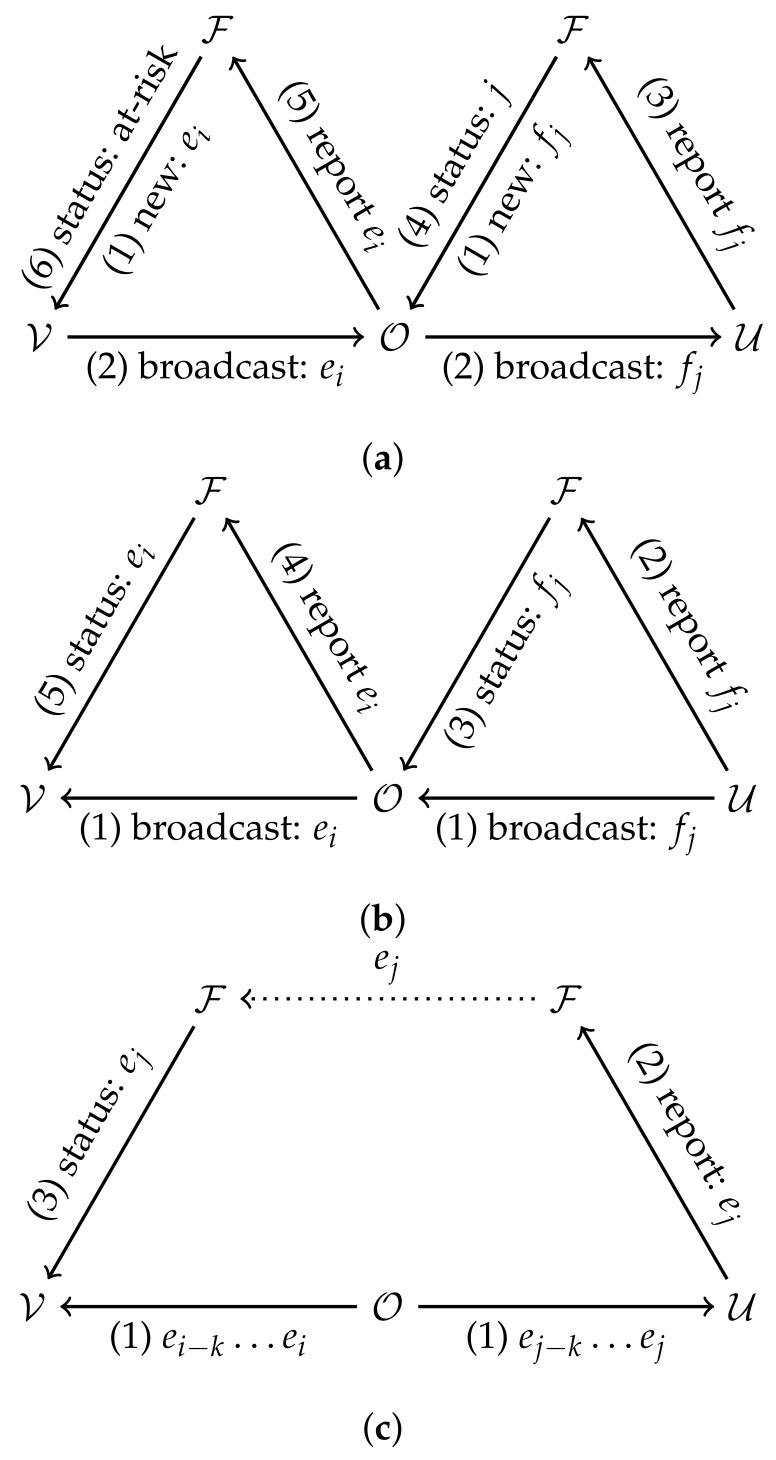
Ephemeral identifier CT with IoT devices. (**a**) Direct and centralized: (1) FE F affects new identifiers to UE V and OE O. (2) O records contact with V and UE U records contact with O. (3) U is diagnosed, reports incoming identifiers containing $f_{j}$ to F. (4) F alerts O of at-risk status at time *j*. (5) O reports identifiers $e_{i}$ received in a short interval after time *j*. (6) F notifies V of risk status. (**b**) Direct and decentralized: (1) OE O records contact with UE U and UE V records contact with O. (2) U is diagnosed, reports $f_{j}$ to FE F. (3) F alerts O of new status of identifiers $f_{j}$. (4) O evaluates its risk and reports $e_{i}$ broadcasted in a short interval after receiving $f_{j}$ as at-risk. (5) F forwards $e_{i}$ to V who computes its risk. (**c**) Indirect and decentralized: (1) OE O broadcasts rolling window of ids to UEs. (2) UE U is diagnosed, reports $e_{j}$ to FE F. (3) F sends $e_{j}$ to UE V that correctly identifies risk status if i−j<k.

There are many ways such O2P contacts can be recorded, we present them below and summarize them in Figure 2.

### 4.1. Ephemeral Identifiers in IoT-CTS

We now describe how the above framework may be adapted with the introduction of IoT devices. We only consider O2P contacts since P2P contacts can be traced in the same way as described above. In the IoT-CTS setting, the list of contacts between UEs is augmented by the second-degree contacts through OEs. Such O2P contacts must account for the duration that the virus stays active in the air or on surfaces. Two UE that had a contact with the same OE in close proximity in time will possibly have recorded different identifiers. The entity that computes risk (the UE in the decentralized model and the FE in the centralized model) must account for this possibility.

#### 4.1.1. Case 1: The Direct Model

In the direct model, both UEs and OEs store incoming and outgoing identifiers. The OE can be alerted of transmission risk by the FEs just as a UE would and forward this alert to other UE it was in contact with through the FE.

In the centralized architecture, the FE can link ephemeral identifiers to OE or UE devices. (1) To report a contact, a diagnosed user uploads its list of received identifiers to the FE. (2) The FE then alerts each OE that was in contact with the diagnosed user. (3) The OE helps forward the risk status by reporting its own list of received identifiers. (4) Based on this list, the FE alerts UEs that were in contact with the OE of their risk.

In the decentralized architecture, the risk status is computed by the UEs. (1) A diagnosed UE would upload their list of outgoing identifiers to the FE. (2) OEs would download that list and check for a registered contact. (3) The OEs with positive checks would then upload the list of outgoing identifiers to the FE. (4) Other UEs can then download the list of OE identifiers and compute their risk status based on this information.

#### 4.1.2. Case 2: The Indirect Model

In the indirect model, OEs passively emit identifiers, but do not collect UE identifiers. Since OEs do not store lists of incoming identifiers, the entity that evaluates risk (FE or UE) must have access to the diagnosed UE’s list of O2P contacts. However, if the OE identifiers are time-evolving, and since the novel coronavirus can remain in suspension or on surfaces for some time, potential second-hand contacts will be missed through this approach. This can be remediated by having OEs broadcast a *rolling window* of identifiers. For example, at any time the OE broadcasts the identifiers of the past hour. An indirect contact through the OE can be identified by checking the intersection of logged OE identifiers. The exact length of the window can be determined by the lifespan of the virus in the air and on surfaces.

The indirect model is incompatible with the centralized architecture. In this architecture, the diagnosed user would upload its list of incoming UE identifiers and the window of OE identifiers for each OE contact. The FE could identify which UE and which OE the diagnosed UE has been in direct contact with, but cannot know which UE has been in contact with the same OE as the diagnosed UE (indirect contact) since this list is stored locally on UE devices.

In a decentralized architecture, (1) the diagnosed UE would upload its list of outgoing identifiers and its list of incoming OE identifiers. (2) Other UEs would download these lists and compare them with their own incoming OE and UE identifiers to compute their risk status.

### 4.2. Privacy Analysis

In classical (non-IoT) CTS, Vaudenay [19] found that centralized systems pose privacy risks for *all users* against the *central authority* and that decentralized systems pose privacy risk for *diagnosed users* against *anyone*. Most privacy issues with CT also apply to IoT-CTS. We identify additional factors relating to privacy and IoT-CTS: IoT devices have no private lives to protect; may be under the control of malicious entities; and are immobile or at the very least their position can be assumed to be known by their operators. We make the following observations on the privacy of IoT-CTS:

1. The *nature* of the privacy risk differs. Whereas P2P contacts reveal information on the social graph, O2P contacts reveal information on the location history of the UE;
2. In the direct model, there is a new privacy risk from diagnosed users against the OEs in the decentralized architecture: they learn the identifiers of diagnosed UEs. For this model, the centralized architecture is better at protecting privacy aginst the OEs;
3. The indirect model provides privacy against OEs, but discloses some location information of the diagnosed UE to other UEs.

Note that CTS based on ephemeral identifiers presented earlier in this section do not completely eliminate the privacy risks, but make privacy attacks harder to execute. More advanced techniques to further improve privacy of CTS solutions can be readily applied to IoT-CTS. *Private set intersection* [20] or *homomorphic encryption* [21] can be used to identify contacts by checking if two lists of identifiers share a common element without disclosing either list. *Diffie–Hellman key exchange* allows users to generate fresh identifiers at each contact that are known only to the two users involved [22,23]. Blockchains have been proposed in several other works to replace trust in a central authority in traditional CTS. For a review of these proposals, see [24,25]. The application of these techniques to IoT-CTS could form the basis of future work.

## 5. Case Study

Let us consider a typical deployment of the proposed IoT-CTS in a public area as illustrated in the geographical layer in Figure 1, where an IoT-CTS solution is assumed to be applied to a downtown area in Toronto where typical public facilities are located, such as bus or subway stations, museums, and shopping malls. Based on the population density and the area 0.6 ${\mathrm{km}}^{2}$, the population size of this area is approximated to 5000.

**Figure 3:**
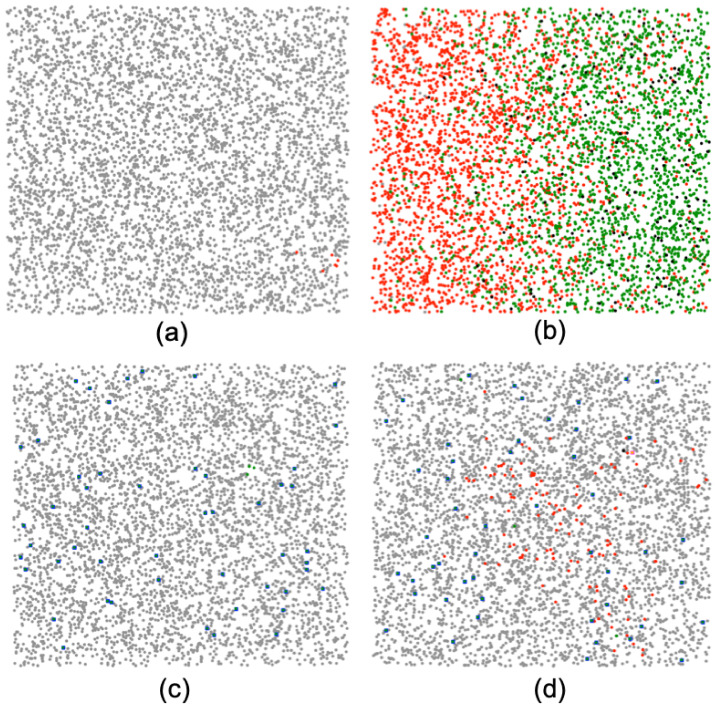
A visualized simulation process, where (**a**,**b**) show the benchmark scenario in the initial and intermediate phases, respectively; and (**c**,**d**) shows the users and objects deployed in the initial and intermediate phases, respectively. User states are indicated in dots with healthy (in gray dots), infectious (in red dots), recovered (in green dots), and fatality (in black dots) states. UEs are shown in blue squares.

A CTS has been known to “flatten the curve”, where containing the infectious disease by reducing the infectious cases. An agent-based Python simulation is used to evaluate such key epidemiological performance. Some main simulation parameters are listed in Table 2, and in our simulation implementation the physical parameters are mapped to generic values. For example, geographical area is mapped to 2×2 plane. UEs and OEs are assumed randomly distributed in the area as a general scenario. CT data are assumed to be processed within an FE, and OEs are assumed to have a generic sensing capability of detecting OE–UE interactions. The basic sensing technology for OEs we consider is based on proximity sensing, which is a representative sensing mechanism adopted by current CTS solutions. The benchmark CTS for comparison is, therefore, based on the BLE proximity sensing, which has been broadly adopted in most countries through 2021 [15]. In our simulations, each user moves at the 1.8 or 2.35 m/s average speed with the consideration of other real-world factors, including age risk, healthcare capacity, and mortality rate based on the recent public health information. The detection range [26] of OEs based on proximity sensing and environment sensing are 2 m and 20 m, respectively. The first infectious case is randomly introduced on the third day. The example processes of the simulations are visualized in Figure 3.

**Figure 4:**
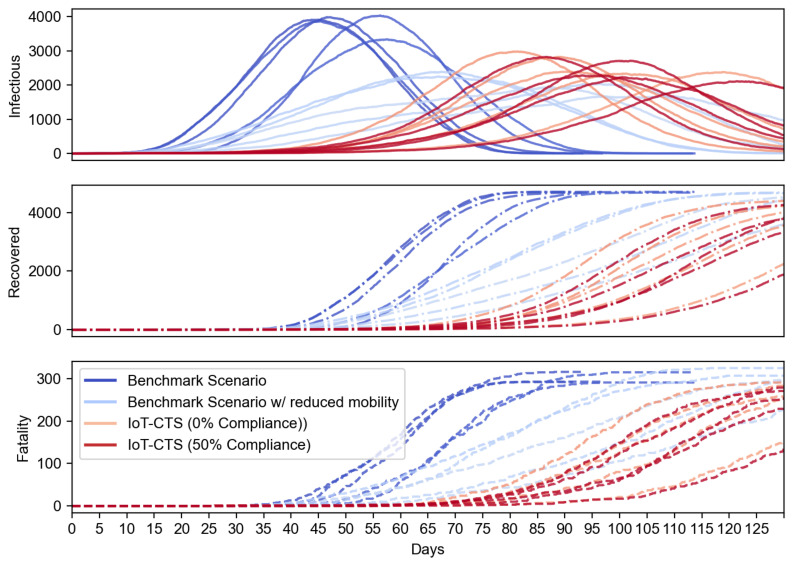
Epidemiological performance between benchmark solutions and IoT-CTS solutions.

We first evaluate how the proposed IoT-CTS performs compared to the existing benchmark CTS solutions based on proximity sensing. In Figure 4, the benchmark scenario represents the current CTS solution. To evaluate our IoT solution in a strict condition, we use the modified benchmark scenario where population mobility is reduced to 50%. In our IoT-CTS solutions, users are informed of the risk of exposure to an infectious disease from proximity (<2 m) to each other and contacts from contaminated surfaces. We use $N_{OE}=50$ in the proposed IoT-CTS solutions and experiment with two scenarios: one is individuals do not practice a relocation strategy (e.g., users go to a confined location with reduced mobility) when the risk of exposure is detected, marked as 0% compliance, and the other is 50% of individuals practice relocation strategy, marked as 50% compliance. The results shown in Figure 4 indicate that our IoT-CTS solution outperforms the benchmark scenarios as all curves are flattened even if the population mobility is significantly reduced in the benchmark scenario. We can see that the recovered and fatality population are reduced with the IoT-CTS solution compared to benchmark scenarios. The infectious population of our IoT-CTS solutions is slightly over the benchmark scenario with reduced mobility. However, we should note that contaminated object surfaces are introduced in the IoT-CTS scenarios, while such objects are not present in the benchmark scenarios.

**Figure 5:**
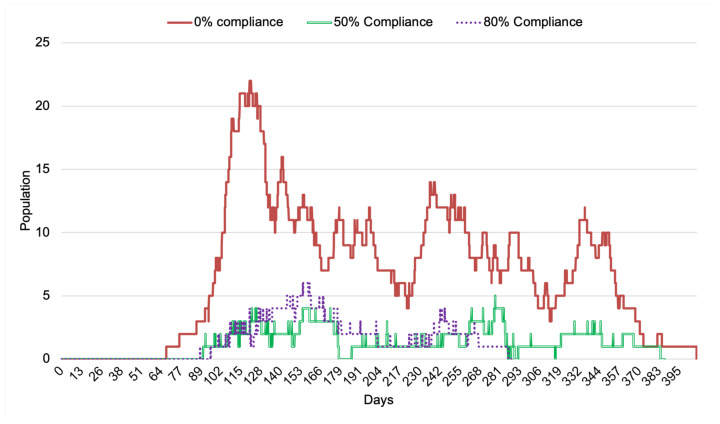
Compliance rate of population versus infectious cases through contaminated surfaces (s=1.8 m/s, $N_{OE}=50$).

Now we examine with IoT-CTS how the compliance rate of relocation strategy affects the performance of infectious cases through contaminated surfaces detected by OE. From the results shown in Figure 5, the impact of the compliance rate has an impact on the infectious cases where the number of the infectious population is most when no compliance is adopted. The increasing compliance rate can positively impact the reduction of the infectious population in terms of case total and the duration of the transmission.

**Figure 6:**
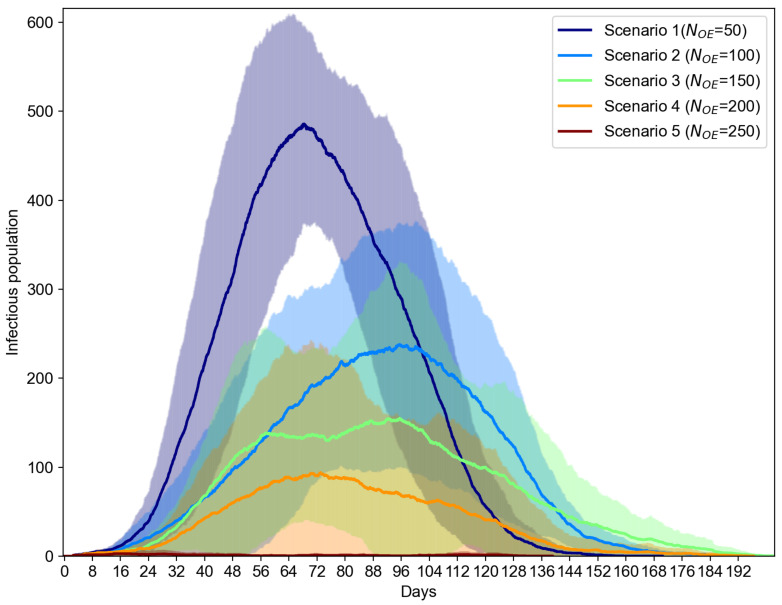
Infectious population vs. $N_{OE}$ (s=2.35 m/s, $N_{OE}=200$).

**Figure 7:**
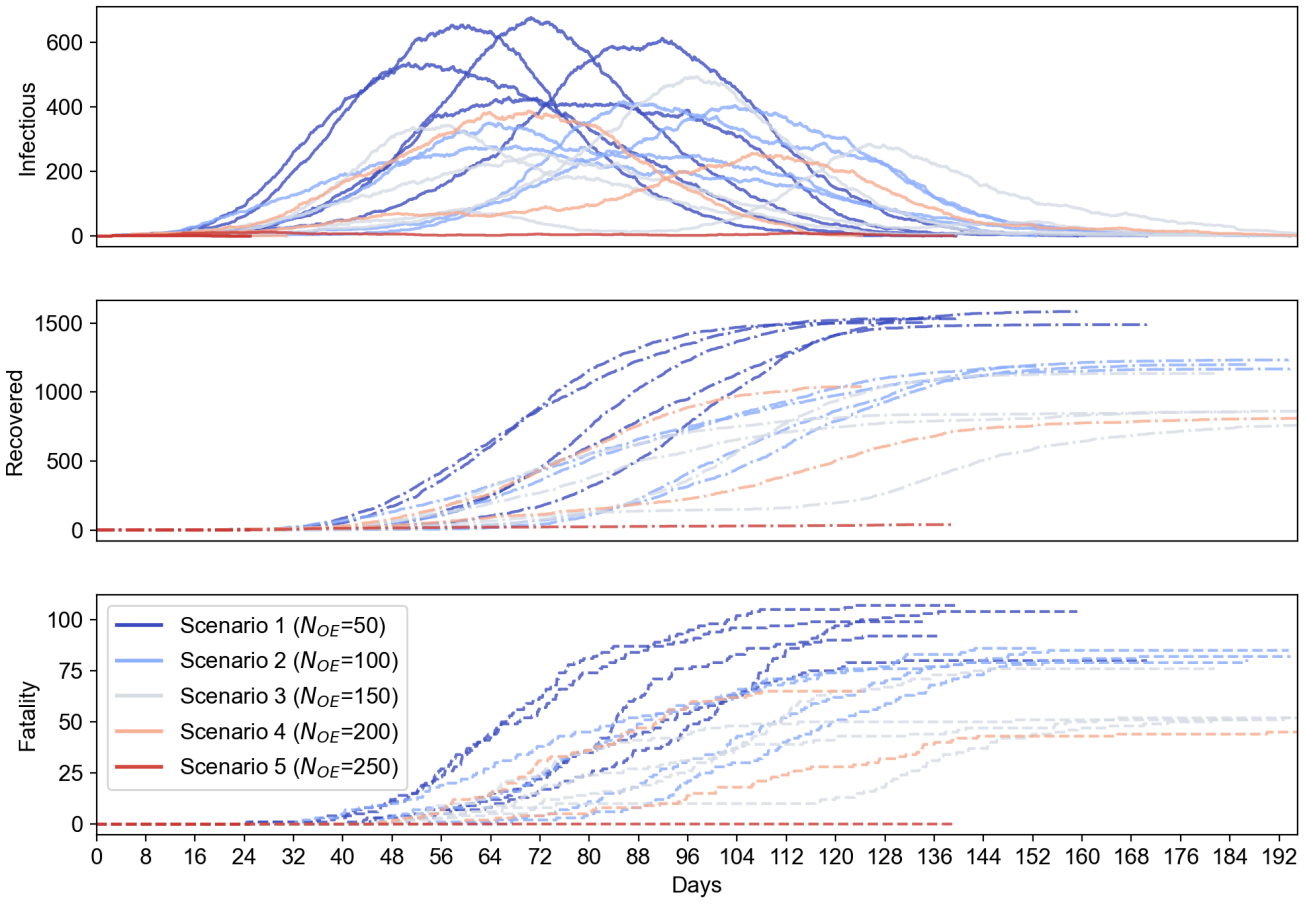
Epidemiological performance vs. $N_{OE}$ (s=2.35 m/s, $N_{OE}=200$).

Now, let us see how the use of OEs can impact the O2P scenarios in terms of the infectious population in an extended IoT-CTS case, where we propose the combined use of two typical kinds of OEs in this IoT-CTS case. The OEs based on the environment sensing (as shown in Table 1 and depicted in Figure 1) is proposed to add to the evaluation because they can directly sense the airborne viruses in a surrounding environment which cannot be performed with only proximity sensing. We also set a certain ratio of the environment sensors to 20% based on practical deployment considerations, where bio-sensing-based environment sensors are relatively more expensive than the proximity sensors and should be used in a small portion of the total OEs. Additionally, we let $T_{d}=1$ for OEs where contaminated OEs become disinfect every day, which can reflect the real-life cases where object surfaces are disinfected at a certain frequency. In this setup, the environment sensor-based OEs and proximity sensor-based OEs are randomly deployed where the total number of OEs, $N_{OE}$, ranges from 50 to 250. The simulation results are shown in Figure 6, where the mean value and error bars based on standard error are plotted as shaded area for each scenario. From Figure 6, we can see the use of environment sensor-based OEs can help reduce the number of infectious population. The increasing number of environment sensor-based OEs can significantly reduce the infectious cases for all OE sizes. From Figure 7, the proposed IoT-CTS solution with increasing number of environment sensors and proximity sensors can effectively flatten the curves for the recovered and fatality population as well.

**Figure 8:**
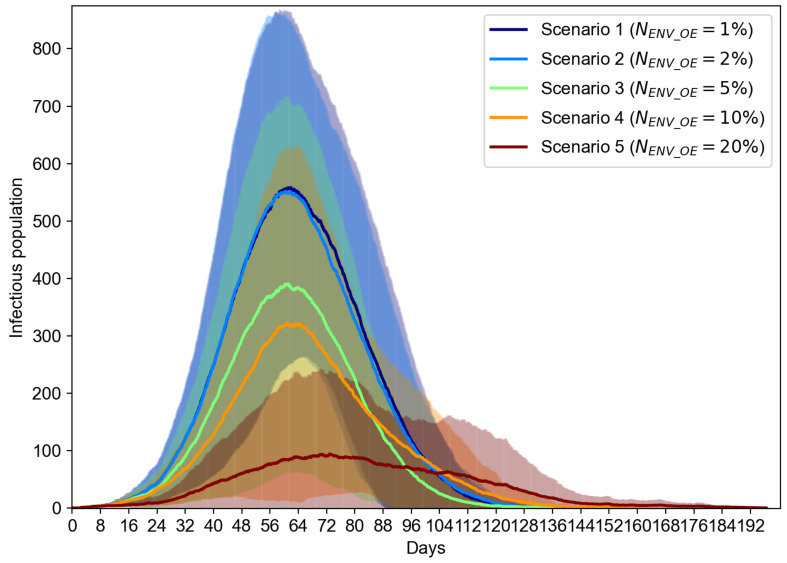
Infectious population vs. the number of environment sensors ($N_{ENV\_OE}$) (s=2.35 m/s, $N_{OE}=200$).

**Figure 9:**
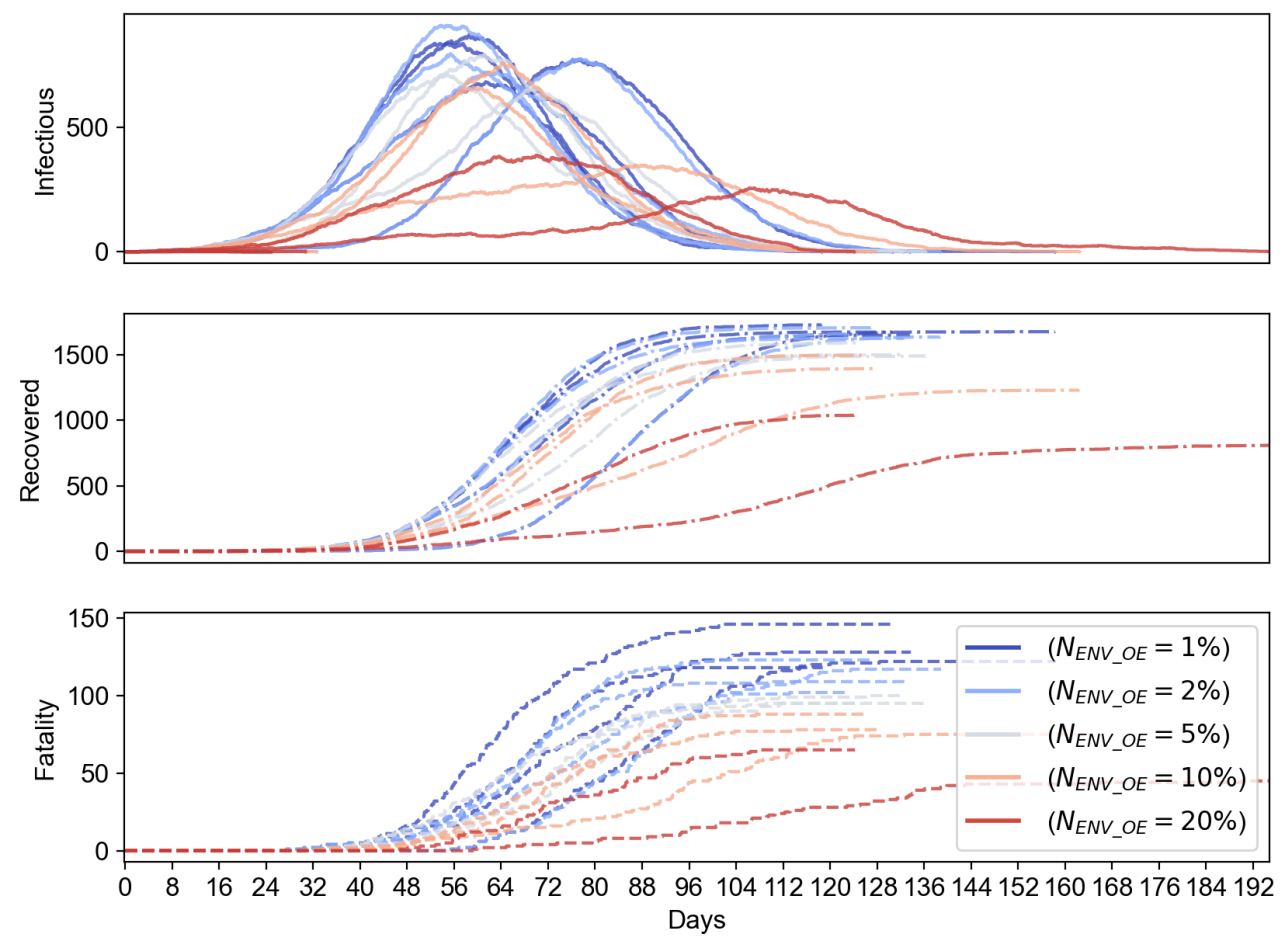
Epidemiological performance vs. the number of environment sensors ($N_{ENV\_OE}$) (s=2.35 m/s, $N_{OE}=200$, $T_{d}=1$).

To evaluate the effect of the portion of environment sensor-based OEs, we evaluate another case where $N_{ENV\_OE}$ can range from 1% to 20%, while we keep the total number of OEs to be 200. The results shown in Figure 8 suggest that the increasing number of environment sensor-based OEs can overall reduce the number of infectious population. We can notice that the reduction of the infectious cases is marginal when $N_{ENV\_OE}$ increases from 1% to 2%, but starts to be significant when $N_{ENV\_OE}$ changes from 10% to 20%. From the results in Figure 9, we can see that the increasing percentage of environment sensors in an IoT-CTS solution can improve the epidemiological performance and flatten the curves of the infectious, recovered, and fatality population.

We have shown the effectiveness based on the typical scenarios of P2P and O2P where UEs and OEs are used. We can see that the relocation strategy is suggested to be applied as a self-directed strategy or measure, where individuals can opt to move to the specific area (such as home or quarantine place) when they are informed of risk of exposure to a coronavirus, e.g., SARS-CoV-2. Additionally, the case study with our extensive simulations suggests that the combined use of sensors in IoT-CTS based on environment sensing and proximity sensing are effective. These scenarios aim to evaluate generic IoT-CTS solutions, and additional measures based on the adoption of IoT-CTS solutions are expected to improve the performance further.

## 6. Future Directions

Considering the increasing interests and need for additional research efforts based on CT solutions from the research community to combat current COVID-19 variants and future infectious diseases, we would like to discuss some open problems for research contributions, which are described in the following.

### 6.1. Simulation Platforms

A simulation platform can help save the cost of post-deployment evaluation efforts and examine performance metrics and parameters in the design phase. For example, a discrete-event based simulation package can reveal the system dynamics that cannot be fully discovered from an epidemiological model. It can incorporate real-world factors into consideration, such as individual behaviors, user adoption, P2P/O2P interactions, sensing performance, privacy risks, and protocols.

### 6.2. Sensing Systems

Sensing systems for IoT-CTS need enhancements to a greater extent. For example, the usability and miniaturization, and sensing capability on an IoT device with OE/UE entities can be improved. The precision, speed, and stability for at-scale deployments need to be enhanced. Intelligent sensing and processing of data from various sources with artificial intelligence (AI) are another area to be studied.

### 6.3. Data Management and Privacy

Data management and privacy are key to the IoT-CTS application, which may need to be deeply integrated with tasks handling regular and sensitive CT data. The integration models for functional and data privacy requirements still have much room left for further exploration.

### 6.4. Integration of Underlying Infrastructures

With the expectation that an IoT-CTS is to be deployed on underlying digital infrastructures, how to integrate the IoT-CTS with the IT infrastructures or services, such as cloud services, software-ized networking, mobile networks, and CDN, need to be resolved. For example, edge computing may provide infrastructural support for the essential interactions and communication between IoT-CTS entities, facilitating entity interactions, data processing, and computation offloading with adjacent FEs on a cloudlet. In addition, how to utilize various data sources also leads to an open issue.

### 6.5. Hardware and Software Support, Integration, and Optimization

IoT-CTS software support for optimal communication horizontally between entities and vertically across layers with underlying networks or computing nodes, requires further development. Additionally, an effective IoT-CTS depends on the seamless integration and optimization of the hardware and software components. For example, the architectural entities need to be operated in a platform using low-power and high-precision sensors integrated with an optimized software framework.

## 7. Conclusions

An IoT-CTS offers appealing advantages over conventional CT solutions: it works with essential sensing, networking, and computing technologies in a scalable setup for the COVID-19 and similar infectious diseases. With the proposed architecture and solution, an IoT-CTS has shown to be effective with self-directed strategies and with the use of OEs, which may be applied together with public health measures. The IoT-CTS architecture aims to provide effective CT solutions with comprehensive coverage of contact events, enable synergistic system designs, and promote integrated and standardized implementations for evolution. The introduction of networked IoT devices in CTS adds new privacy challenges. Decision-makers should weigh the public health benefits of CTS against the privacy risks, and consider more advanced privacy-protection measures. This article has systematically explored the combined use of various sensing technologies to help design better CT solutions to combat current and future infectious diseases. 

## Figures and Tables

**Table 1:** Types of sensing technologies for IoT-CTS.

Type	Example Devices	P2P	O2P	Automatic	Speed	Precision
Location-based	GPS, Mobile [5,7,11]	✓	×	✓	Medium, High	Medium, High
Distance	Ultrasonic, Magnetometer [4]	✓	✓	✓	High	Medium, High
Vision	Camera	✓	✓	✓	High	Medium, High
Vision	QR code reader	✓	✓	×	Low	High
Touch	Inductive/capacitive sensors	×	✓	✓	High	High
Proximity	Bluetooth/BLE [5,11,15]	✓	✓	✓	High	High
Touch, Proximity	NFC, RFID	✓	✓	✓	High	Low, Medium
Environment	Biosensor [16]	×	✓	✓	Low, Medium	Low, Medium

**Table 2:** Simulation parameters.

Parameters	Value	Unit
Deployment plane size	780 × 780	m${}^{2}$
Population density per km${}^{2}$	8210	–
Infection probability	5%	–
Population size	5000	–
Mean population age	45	–
Age risk increase range	[55, 75]	–
Healthcare capacity	14	beds/1000 ppl.
Infection range between UEs	2	m
Detection range of OEs (proximity sensing)	2	m
Detection range of OEs (environment sensing)	20	m
Average walking speed (*s*)	{1.8, 2.35}	m/s
Mortality probability	0.021	–
Max. mortality prob. for older age	0.1	–
Recovery duration	[20, 30]	days
Number of OEs ($N_{OE}$)	[50, 250]	–
Surface sanitizing duration ($T_{d}$)	1	day
